# Supplementary material for: Copper catalysis at operando conditions—bridging the gap between single nanoparticle probing and catalyst-bed-averaging
Source: Nat Commun. 2020 Sep 24;11:4832. doi: 10.1038/s41467-020-18623-1 (PMC7518423; doi:10.1038/s41467-020-18623-1)
Supplement: Supplementary file 1 — Supplementary Information [file 41467_2020_18623_MOESM1_ESM.pdf]

Supplementary Information

Copper Catalysis at Operando Conditions - Bridging the Gap between Single Nanoparticle Probing and Catalyst-Bed-Averaging

Albinsson et al.

Supplementary Methods

Nanofluidic reactor fabrication

Outlined below are the fabrication steps for the nanofluidic chip that are also illustrated in **Supplementary Figure 2**.

Fabrication of alignment marks
(a) Spin coating HMDS adhesion promoter (MicroChem) at 3000 rpm for 30 sec and soft baking on a hotplate (HP) at 115˚C for 120 sec. Spin coating UV5 (MicroChem) at 3000 rpm for 60 sec and soft baking (HP) at 130˚C for 120 sec. (b) Electron-beam exposure of alignment marks for both optical and electron-beam lithography at 10 nA with a shot pitch of 24 nm and 25 $\mu C/cm^2$ exposure dose. (c) Post-exposure bake (HP) at 130˚C for 90 sec. (d) Development in MF-24A (Microposit) for 90 sec, rinsing in water and drying under N_2-stream. (e) Reactive-ion etching (RIE) for 15 sec at 60 mTorr chamber pressure, 60 W RF-power, 60 sccm O_2-flow (descum). RIE for 30 min at 40 mTorr chamber pressure, 50 W RF-power, 100 W ICP-power, 50 sccm Cl_2-flow (1200 nm etch depth in silicon).

Thermal oxidation
(a) Cleaning in 50 mL H_2O_2 + 100 mL H_2SO_4 at 130˚C for 10 min, rinsing in water and drying under N_2-stream. (b) Wet oxidation in water atmosphere for 45 min at 950˚C (200 nm thermal oxide).

Nanofluidic channels
(a) Spin coating HMDS adhesion promoter (MicroChem) at 3000 rpm for 30 sec and soft baking on a hotplate (HP) at 115˚C for 120 sec. Spin coating UV5 (MicroChem) at 3000 rpm for 60 sec and soft baking (HP) at 130˚C for 120 sec. (b) Electron-beam exposure of nanofluidic structures at 10 nA with a shot pitch of 24 nm and 25 $\mu C/cm^2$ exposure dose. (c) Post-exposure bake (HP) at 130˚C for 90 sec. (d) Development in MF-24A (Microposit) for 90 sec, rinsing in water and drying under N_2-stream. (e) Reactive-ion etching (RIE) for 5 sec at 50 mTorr chamber pressure, 50 W RF-power, 50 sccm O_2-flow (descum). RIE for 2.5 min at 40 mTorr chamber pressure, 50 W RF-power, 100 W ICP-power, 50 sccm NF_3-flow (100 nm etch depth in SiO_2).

Microchannels
(a) Spin coating HMDS at 3000 rpm for 30 sec and soft baking (HP) at 115˚C for 2 min. Spin coating S1813 (Shipley) at 3000 rpm for 30 sec and soft baking (HP) at 115˚C for 2 min. (b) Expose microchannels for 8 sec in contact aligner at 6 mW/cm^2 intensity. (c) Development in MF-319 (Microposit) for 60 sec, rinsing in water and drying under N_2-stream. (d) Buffered oxide-etch for 2 min to remove thermal oxide, rinsing in water and drying under N_2-stream. (e) Deep reactive-ion etching at 6 mTorr chamber pressure, 800 W RF-power, 8 W platen power, 130 sccm SF6-flow (Si-etch), and of 5 sec at 6 mTorr chamber pressure, 800 W RF-power, 8 W platen power, 85 sccm C4F8-flow (passivation) at a rate of 600nm/cycle. (f) Removal of resist in 50 mL H_2O_2 + 100 mL H_2SO_4 at 130˚C for 10 min, rinsing in water and drying under N_2-stream. The resulting channels have a depth of 60 μm measured using a Dektak 150 surface profiler.

Inlets (from backside)
(a) Magnetron-sputtering of 200 nm Al (hard mask). (b) Spin coating S1813 at 3000 rpm for 30 sec and soft baking (HP) at 115˚C for 2 min. (c) Expose inlets for 10 sec in contact aligner at 6 mW/cm^2 intensity. (d) Development in MF-319 for 60 sec, rinsing in water and drying under N_2-stream. (e) Aluminum wet etch (H_3PO_4:CH_3COOH:HNO_3:H_2O (4:4:1:1)) for 10 min to clear the hard mask at inlet positions. (f) Deep reactive-ion etching for 300 cycles of 12 sec

at 5 mTorr chamber pressure, 600 W RF-power, 10 W platen power, 130 sccm SF_6-flow (Si-etch), and of 7 sec at 5 mTorr chamber pressure, 600 W RF-power, 10 W platen power, 85 sccm C_4F_8-flow (passivation) at a rate of 2 µm/cycle. (g) Removal of Al-hard mask in 50 mL H_2O_2 + 100 mL H_2SO_4 at 130˚C for 10 min, rinsing in water and drying under N_2-stream.

Heater elements on the backside
(a) Spin coating HMDS at 3000 rpm for 30 sec and soft baking (HP) at 115˚C for 2 min. Spin coating LOR3A (MicroChemicals) at 3000 rpm for 30 sec and soft baking (HP) at 180˚C for 5 min. Spin coating S1813 (Shipley) at 3000 rpm for 30 sec and soft baking (HP) at 115˚C for 2 min. (b) Expose heater elements with direct-laser lithography at 6 mW/cm^2 intensity. (c) Development in MF-319 (Microposit) for 60 sec, rinsing in water and drying under N_2-strea m. (d) Electron-beam evaporation of 10 nm Cr / 100 nm Pt. (e) Lift-off in remover Rem400 (MicroChemicals), rinsing in isopropanol, and drying under N_2-stream.

Nanoparticles inside nanoreactors
(a) Spin coating Copolymer MMA(8.5)MMA (MicroChem Corporation, 10 wt % diluted in anisole) at 6000 rpm for 60 sec and soft baking (HP) at 180˚C for 10 min. Spin coating PMMA A2 at 3000 rpm for 60 sec and soft baking (HP) at 180˚C for 10 min. (b) Electron-beam exposure at 1 nA with a shot pitch of 2 nm and 2000 µC/cm2 exposure dose. (c) Development in methyl isobutyl ketone : isopropanol (MIBK:IPA) (1:3) for 120 sec, rinsing in isopropanol and drying under N2-stream. (d) Electron-beam evaporation of 40 nm Cu. (e) Lift-off in acetone, rinsing in isopropanol, and drying under N2-stream.

Fusion bonding
(a) Cleaning of the lid (175 µm thick 4”-pyrex, UniversityWafers) in H_2O:H_2O_2:NH_3OH (5:1:1) for 10 min at 80˚C. (b) Pre-bonding the lid to the substrate by bringing surfaces together and manually applying pressure. (c) Fusion bonding of the lid to the substrate for 5 h in N_2 atmosphere at 550˚C (5˚C/min ramp rate).

Dicing of bonded wafers
Cutting nanofluidic chips from the bonded wafer using a resin bonded diamond blade of 250 µm thickness (Dicing Blade Technology) at 35 krpm and 1 mm/s feed rate.

Estimated Catalyst surface area

To get a rough estimate of the total exposed catalytic surface area inside each of the reactors the particles were assumed to be identical cylindrical disks. The total exposed Cu surface area was estimated as: $A_{\mathrm{surf}} = n_{\mathrm{particles}} \times (\pi r_{\mathrm{Cu}}^2 + 2\pi r_{\mathrm{Cu}} h_{\mathrm{Cu}}) \approx 26\ \mu m^2$, where n = 1000 is the number of particles, r_{Cu} = 60 nm is the Cu particle radius and h_{Cu} = 40 nm is the Cu particle height.

Finite-volume simulations

To verify the anticipated different reactant concentration profiles in the two reactor geometries, we simulated the O_2 concentration profiles created during the CO oxidation reaction over the Cu nanoparticles for both reactor geometries using finite-volume simulations (**Figure 2a)** For the well-mixed design, due to the relatively low flow speed in the reaction chamber, we find that diffusion is effectively counteracting concentration gradients resulting from the CO oxidation reaction on the Cu particles (**Figure 2 a,c**). Hence all 100 particle patches experience close to identical local O_2 concentration (less than 7 % variation, calculated by evaluating C_i / C_j, where C_i is concentration on patch *i*). In contrast, due to the higher flow speed in the plug-flow reactor in combination with reactant conversion, each patch experiences significantly different O_2 concentration, with a variation of 72 % between the first and the last patch (**Figure 2b,c**).

Method

Finite-volume simulations were performed to simulate the spatial distribution of O_2 inside the well-mixed and the plug-flow reactors. In these simulations, the gas mixture passes through a three-dimensional computational domain representing the reaction zone along with the nanochannel segments that connect it to the upstream and downstream micrometer-sized segments for fast gas exchange (cf. **Figure 1b**). The coupled system of partial differential equations used to describe the gas flow, and the tracer transport and reaction, is discretized in a finite-volume framework and solved using the computational fluid dynamics (CFD) code ANSYS Fluent 2019 R3. The flow is assumed to slip freely over the surfaces and the flow rate was set to 1.5×10^{12} s^{-1} based on the experimental results. On top of this flow field, a convection-diffusion-reaction equation is solved for a tracer species (representing O_2), the diffusivity of which is set equal to the Knudsen diffusivity of the nanochannel:

$$\nabla \cdot (\rho \mathbf{u} Y) = \nabla \cdot (\rho D_{Kn} \nabla Y) \quad (1)$$

Here, ρ is the gas phase density (kgm^{-3}), **u** is the velocity vector (m/s), Y is the mass fraction of the tracer species, and D_{Kn} is the Knudsen diffusivity (m^2s^{-1}). The boundary condition for Y is a mass fraction of 0.001 at the inlet and zero flux on all walls, except at the location of each of the 100 nanoparticle patches. At these locations, the applied boundary condition is that the flux towards the boundary is balanced by a first-order surface reaction at the wall:

$$\left.\frac{\partial Y}{\partial n}\right|_{w} = \frac{kY_w}{D_{Kn}} \quad (2)$$

Here, n is the direction of the wall surface normal, k is an effective reaction-rate constant for the surface reaction (discussed below) (m/s), and subscript w refers to values at the wall. Variations of Y inside the reactor are assumed not to influence the fluid properties, so that the velocity field is unaffected by the conversion of the reactants. In this way, the local conversion relative to the chosen inlet mass fraction can be obtained at any given point inside the domain.

The parameter k is to be interpreted as an effective global surface reaction-rate constant where the intrinsic reaction kinetics are approximated by a single-step first-order reaction. The single-step reaction can then be thought of as representing the rate-limiting step of a more complex mechanism while assuming a first-order dependence on the reactant (tracer) concentration. In the event that a distinct rate-limiting step is indeed of first order, k would be defined as:

$$k = AfS\exp\left(-\frac{E_a}{RT}\right) \tag{3}$$

where A is the Arrhenius frequency factor, f is a function describing the dependence of the reaction rate on the surface coverages, S is a parameter relating the wall surface of the 10 x 10 nanoparticle array to the actual number of active sites represented by the 100 nanoparticles, and $\exp\left(-\frac{E_a}{RT}\right)$ is the Arrhenius reaction probability term. If a single rate-limiting step cannot be unambiguously identified, k becomes a lumped parameter that characterizes the global rate of conversion for the complete mechanism cast in the form of a single-step first-order reaction. Consequently, k becomes a model parameter that represents the overall rate of reaction and should be chosen so the global conversion over the reactor matches the one observed experimentally. When that criterion is fulfilled, the detailed finite-volume simulation results can be used to obtain spatially resolved insight into the combined effects of reaction and transport inside the reactor on the concentration fields.

Pressure profile calculations

As we have previously observed,[1] classification of the gas flow across the nanofluidic chip is non-trivial because the system encompasses continuum flow in the upstream channel (Kn $\rightarrow$ 0), free molecular flow in the downstream channel approaching the exit to the mass spectrometer (Kn $\rightarrow \infty$), and slip and transition flow in between (intermediate Kn regimes). Here, we again used the unified channel flow model of Beskok & Karniadakis,[2] in which the system is treated as a series of connected segments of differing dimensions, to calculate the pressure profiles across the full system. The pressures at the junctions between each segment can be computed by observing that the steady-state mass flow rate through the system must be constant for continuity and solving the resulting system of nonlinear equations across each segment with the known inlet and outlet pressures. A full pressure profile can then be obtained using the implicit relation for pressure provided by Beskok & Karniadakis[2] and the profiles for the two reactors studied here are shown in **Supplementary Figure 4**. The parameters in the unified flow model were specified as in our previous work, with the slip coefficient set to b = -1 and the tangential momentum accommodation coefficient set to unity. The coefficient α was determined using the analytical fit function provided in the original reference.[2] Minor losses were neglected.

Local concentration estimation

Based on the simulated concentration profiles (**Figure 2**) we can calculate the local O_2 concentration experienced by the particles as they start to oxidize as:

$$C_{\mathrm{local}} = C_{\mathrm{inlet}} \times X \tag{4}$$

where C_{local} is the local concentration inside the reactor, C_{inlet} is the concentration at the inlet of the reactor and X is the local relative concentration extracted from the simulations (**Figure 2c**). Using eq. 1 and the observation that all particles in the batch reactor oxidize at an inlet O_2 concentration of 0.3 %, the local O_2 concentration at the start of Cu oxidation is 0.072 %. In comparison, the same calculation applied to the first array of the plug-flow reactor, which oxidized at a concentration of 0.12 %, corresponds to a local concentration of 0.074 %.

Microkinetic model

A microkinetic model was developed to simulate the reaction kinetics in both the well-mixed and plug-flow reactors.

Elementary reaction steps

The elementary reactions considered in the model are listed in **Supplementary Table 1**.

Supplementary Table 1. Elementary reaction steps included in the microkinetic model. Abbreviations shown in brackets are used as subscripts to identify the corresponding rate constants in the text. Active sites are indicated with an asterix (*) and O_{ox} represents sites deactivated by oxide formation.

Adsorption and desorption (ads/des)	$\mathrm{CO} + * \leftrightarrow \mathrm{CO}*$<br>$\mathrm{O_2} + * \leftrightarrow \mathrm{O_2}*$	(R1)<br>(R2)
Dissociation and recombination (dis/rec)	$\mathrm{O_2}* + * \leftrightarrow 2\mathrm{O}*$	(R3)
Oxidation (rxn)	$\mathrm{CO}* + \mathrm{O}* \rightarrow \mathrm{CO_2} + 2*$	(R4)
Oxide formation (ox)	$\mathrm{O}* \rightarrow \mathrm{O_{ox}}$	(R5)
Oxide reaction (rox)	$\mathrm{CO}* + \mathrm{O_{ox}} \rightarrow \mathrm{CO_2} + 2*$	(R6)

The adsorption reactions R1 and R2 were assumed to be barrierless and the rate constants for adsorption and desorption were calculated as in Filot.[3] The reaction rate constants for R3 and R4 were computed from

$$k_i = \nu_i \exp\left(-\frac{\Delta G_{\mathrm{a},i}}{RT}\right), \tag{5}$$

where ν_i is the prefactor for step i, $\Delta G_{\mathrm{a},i}$ was computed from the activation energies on copper in Falsig *et al.* [4], R is the universal gas constant and T is the temperature. CO_2 desorption was assumed to be fast and irreversible.

Reactions R5 and R6 were introduced to account for formation and breakdown of the oxide, as in Zhdanov and Kasemo.[5] The rate constants for oxide formation (R5) and reaction (R6) were chosen such that the predicted main onset of oxidation occurs at the same inlet oxygen concentration and the predicted conversion on the deactivated surface is a similar fraction of the maximum conversion as in the experiments for the well-mixed system. These parameters were then used to model the plug-flow system as well.

Surface dynamics

The rate of change of coverage of each species is modelled on the catalyst surface as follows:

$$\frac{d\theta_{\mathrm{CO}}}{dt} = k_{\mathrm{ads,CO}}\theta_* P_{\mathrm{CO}} - k_{\mathrm{des,CO}}\theta_{\mathrm{CO}} - k_{\mathrm{rxn}}\theta_{\mathrm{CO}}\theta_{\mathrm{O}} - k_{\mathrm{rox}}\theta_{\mathrm{CO}}\theta_{\mathrm{ox}} \tag{6}$$

$$\frac{d\theta_{\mathrm{O_2}}}{dt} = k_{\mathrm{ads,O_2}}\theta_* P_{\mathrm{O_2}} - k_{\mathrm{des,O_2}}\theta_{\mathrm{O_2}} - k_{\mathrm{dis}}\theta_{\mathrm{O_2}}\theta_* + k_{\mathrm{rec}}\theta_{\mathrm{O}}^2 \tag{7}$$

$$\frac{d\theta_{\mathrm{O}}}{dt} = 2k_{\mathrm{dis}}\theta_{\mathrm{O_2}}\theta_* - 2k_{\mathrm{rec}}\theta_{\mathrm{O}}^2 - k_{\mathrm{rxn}}\theta_{\mathrm{CO}}\theta_{\mathrm{O}} - k_{\mathrm{ox}}\theta_{\mathrm{O}} \tag{8}$$

$$\frac{d\theta_{\mathrm{ox}}}{dt} = k_{\mathrm{ox}}\theta_{\mathrm{O}} - k_{\mathrm{rox}}\theta_{\mathrm{CO}}\theta_{\mathrm{ox}} \tag{9}$$

$$\theta_* = 1 - \theta_{\mathrm{CO}} - \theta_{\mathrm{O_2}} - \theta_{\mathrm{O}} - \theta_{\mathrm{ox}} \tag{10}$$

Here, $\theta_{.}$ is the fraction of sites that are unoccupied (*), occupied by an adsorbate (CO, O_2, O), or considered to be oxide sites (ox). The oxide formation process is modelled as a transformation of active (metal) sites to inactive (metal oxide) sites. The oxidation is complete when all sites have been deactivated. The active fraction of the catalyst illustrated in **Figure 4e** and **Figure 5d** is defined as $1 - \theta_{\text{ox}}$.

Reactor models

Simplified reactor models were used to simulate the flow behavior in the two reactors concurrently with the surface dynamics. A tanks-in-series model was developed for each system, guided by the concentration profiles obtained in the finite-volume simulations (SI section 1.4) and the two reactor designs.

The well-mixed reactor was modelled as a single continuously stirred tank reactor (CSTR) with the following dynamics

$$\frac{dP_{\text{CO}}}{dt} = \left(P_{\text{CO}}^{\text{in}} - P_{\text{CO}}\right)\tau^{-1} + \sigma\left(k_{\text{des,CO}}\theta_{\text{CO}} - k_{\text{ads,CO}}\theta_{*}P_{\text{CO}}\right) \tag{11}$$

$$\frac{dP_{\text{O}_2}}{dt} = \left(P_{\text{O}_2}^{\text{in}} - P_{\text{O}_2}\right)\tau^{-1} + \sigma\left(k_{\text{des,O}_2}\theta_{\text{O}_2} - k_{\text{ads,O}_2}\theta_{*}P_{\text{O}_2}\right) \tag{12}$$

$$\frac{dP_{\text{CO}_2}}{dt} = \left(P_{\text{CO}_2}^{\text{in}} - P_{\text{CO}_2}\right)\tau^{-1} + \sigma\left(k_{\text{rxn}}\theta_{\text{CO}}\theta_{\text{O}_2} + k_{\text{rox}}\theta_{\text{CO}}\theta_{\text{ox}}\right) \tag{13}$$

where P_j is the partial pressure of species j, τ is the reactor residence time and σ is the conversion factor to scale the reaction rate from per site per second to Pascals per second.

The plug-flow reactor was represented with ten CSTRs in series, with each model reactor encompassing one catalyst patch of 10x10 nanoparticles (**Supplementary Figure 1**). The dynamics in each CSTR have the same form, but the incoming concentration, P_j^{in}, is the outlet from the previous model reactor. The series of CSTRs permits modelling of concentration gradients and local conditions around/on the surface of each patch. In both cases, there is no spatial resolution within each reactor model and these sub-systems are assumed to be homogeneous.

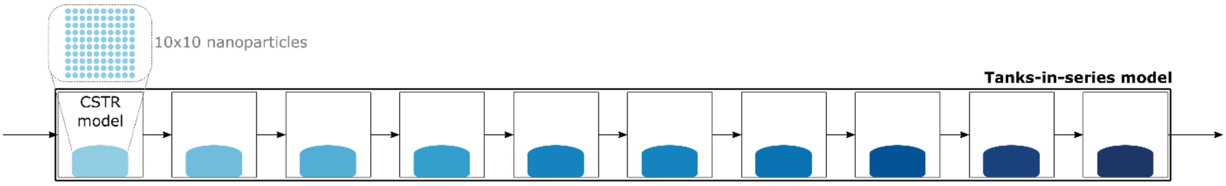

Supplementary Figure 1. **Schematic of the tanks-in-series model for the plug-flow system.** The plug-flow system is represented as a number of well-mixed model reactors in series, with each reactor representing one 10x10 patch of catalyst particles.

The model reactor dimensions are listed in **Supplementary Table 2**. Pressures and flow rates were obtained from unified flow model calculations[2] (SI section 1.5) at 400 °C and a system inlet pressure of 4 bar. As in the experimental work, a fixed concentration of 7% CO in Ar was used, with the O_2 concentration varying in the range 0 – 0.5%. The nanoparticle geometry (assuming the nanoparticles to be cylinders 120 nm in diameter with a height of 40 nm) and the

centre-to-center distance of adjacent Cu atoms were used to estimate a lower bound on the number of active sites per nanoparticle.

Supplementary Table 2. CSTR dimensions and catalyst loading in each reactor model (note that there are ten CSTRs in the plug-flow reactor model, each with the properties shown here).

	Length (μm)	Width (μm)	Height (μm)	Nanoparticles
Well-mixed CSTR	180	120	0.1	1000
Plug-flow CSTRs	21.6	10	0.1	100

Solution method

The system of ODEs describing each reactor/series of reactors was solved using the MATLAB R2018a stiff differential equation solver *ode15s* using backwards differentiation formulas (BDF). Relative and absolute tolerances of 1×10^{-6} and 1×10^{-8} respectively were specified and the 'NonNegative' option was specified for all variables.

Image registration

Individual video frames were stabilized using the image processing toolbox included in Matlab. Specifically, the functions `imref2d,imregtform` and `imwarp` was used to find the x-y translation of each frame compared to the first frame of the movie. Below is a simplified version of the script used to generate the stabilized series of images (`Registered_image{i}`).

```
[optimizer, metric] = imregconfig('monomodal');
optimizer.MaximumIterations = 1e6;
optimizer.MinimumStepLength = 1e-7;
optimizer.MaximumStepLength = 5e-3;
Rfixed = imref2d(size(First_Frame));

for i=2:num_frames
 tformTranslate = imregtform(Frame{i},First_Frame,'translation',optimizer,metric);
 Registered_image{i} = imwarp(Frame{i},tformTranslate,'OutputView',Rfixed);
end
```

where `Frame` contains the original video frames and `First_Frame` is the first video frame.

À trous wavelet filtering for particle detection

The à trous wavelet used for particle detection was implement using the following matlab code (https://gitlab.com/phme/wavelet-tracking) that was adapted from a paper by Olivo-Marin.[6]

```
function [w,A] = a_trous_wavelet(A,J,k)
% Creates the wavelets w of an image A to depth J-1,
% where A(:,:,J) are the smoothed approximations of the original input
% Insignificant coefficients in w are set to zero if <k*MAD
% © P. Messer, 2016

narginchk(1,3)
if nargin==2
    k = 2; % Set k if not specified before
end
w = zeros(size(A));

for i = 1:J-1

    kern = [1/16,zeros(1,2^(i-1)-1),1/4,zeros(1,2^(i-1)-1),3/8,zeros(1,2^(i-1)…
    - 1),1/4,zeros(1,2^(i-1)-1),1/16]; % Convolution Kernel (a trous)
    kernsize = numel(kern);
    % Image gets symmetrical padded to prevent edge effects
    t = conv2(kern,kern,padarray(A(:,:,i),[kernsize kernsize],'symmetric'),'same');
    % Crop image to original size
    A(:,:,i+1) = t(kernsize+1:end-kernsize,kernsize+1:end-kernsize);
    tw = A(:,:,i)- A(:,:,i+1); % Get Wavelet
    mad = abs(tw - median(tw(:))); % Calculate Median Absolute Deviation (MAD)
    sig(i) = k*median(mad(:)); % Set significance level
    tw(tw<sig(i)) = 0; % Remove insignificant wavelet coefficients
    w(:,:,i) = tw;
end
```

For the particle detection, the first wavelet level one (`w(:,:,1)`) was used and peaks were detected by funding local maxima.

Supplementary Discussion

Extended single particle oxidation discussion

To understand the strong dependence of single-particle τ_{ox}-spread within a patch on its position along the catalyst bed, and in particular the observation of the largest single particle τ_{ox}-spread occurring in patches closest to the inlet for both well-mixed and plug-flow reactor, we consider both the spatial and temporal differences imposed by the reactor geometry and the design of the experiment. To start with, the amount of O_2 introduced into the reactor is increased in steps over time. Simultaneously, we observe a gradual deactivation (oxidation) of the catalyst, resulting in fewer and fewer active sites to efficiently convert CO and O_2 to CO_2. As a consequence, the rate of Cu oxidation will be more severely mass-transport limited in the early stages of the experiment. In contrast, the downstream patches, that oxidize at a later stage, will experience a more dramatic "shock" of excess oxygen at a higher concentration once a large fraction of upstream particles has been deactivated by oxidation since the inlet nominal pressure has been increased. Accordingly, the wider τ_{ox} distribution observed for the individual particles in upstream patches can be understood as the consequence of each particle having a unique surface structure (facets), morphology (grains) and other defect abundance, which mediates both the O_2 and CO adsorption affinity and consequentially the activity of the particle,[7,8] as well as the critical local O_2 concentration needed to initiate the bulk oxidation process, in analogy to observations of the hydride formation in individual Pd nanoparticles.[9] As a result, the low abundance of O_2 available for oxidation of Cu in upstream patches close to the inlet results in a slow oxidation that is strongly affected by particle-specific structural properties that dictate the surface coverage of oxygen on each particle.

Finally, looking at a similar analysis for Cu particle consecutive reduction in the flow reactor during the reverted experiment, we find much narrower distributions for the single particle response in each patch and, in contrast to the well-mixed case, a clear position dependence for when it occurs, where the particles closest to the outlet are reduced first (**Supplementary Figure 10b**). This is in good agreement with the general scenario, where the local O_2 concentration is lowered along the channel due to conversion into CO_2, meaning that the condition for reduction is reached first close to the outlet, where the local O_2 concentration is the lowest.

Supplementary Figures

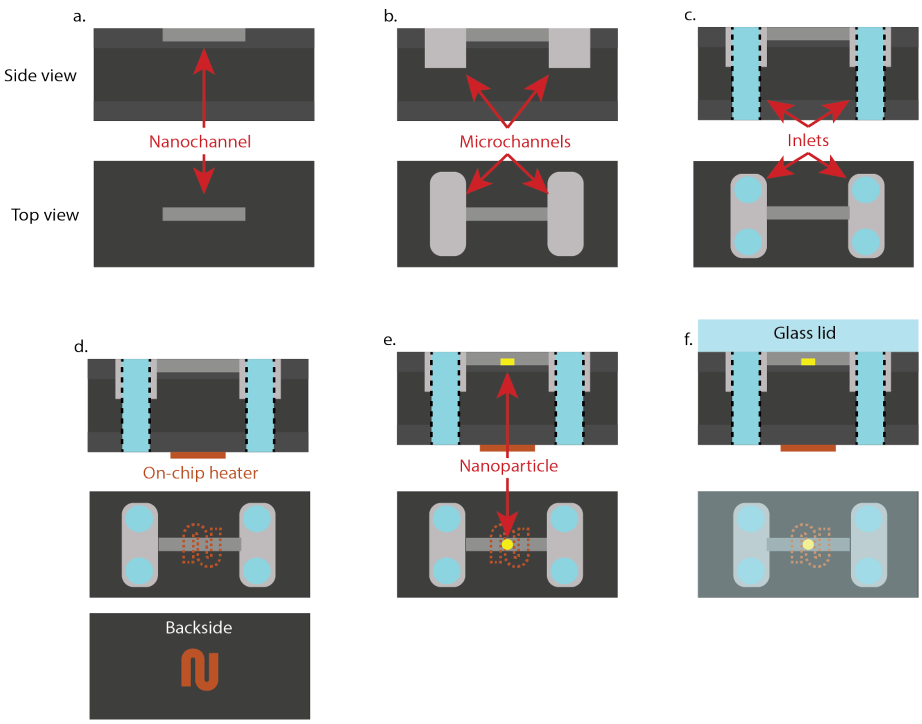

Supplementary Figure 2. Process steps for the nanofabrication of a nanoreactor chip. The starting material is a Si wafer with 200 nm thermal oxide (SiO_2). a) Nanochannels are patterned using EBL and etched using RIE to 100 nm depth in SiO_2. b) Microchannels are patterned using photo-lithography and etched using DRIE through the SiO_2 and in to the Si. c) Holes are patterned using photo-lithography from the backside and etched using DRIE. d) A heater pattern is defined on the backside of the chip using photo-lithography and deposited using electron beam deposition of 10 nm Cr followed by 100 nm Pt. e) Nanoparticles are defined in resist using EBL and deposited using electron beam deposition of the desired material. f) A glass lid is bonded to seal the channels.

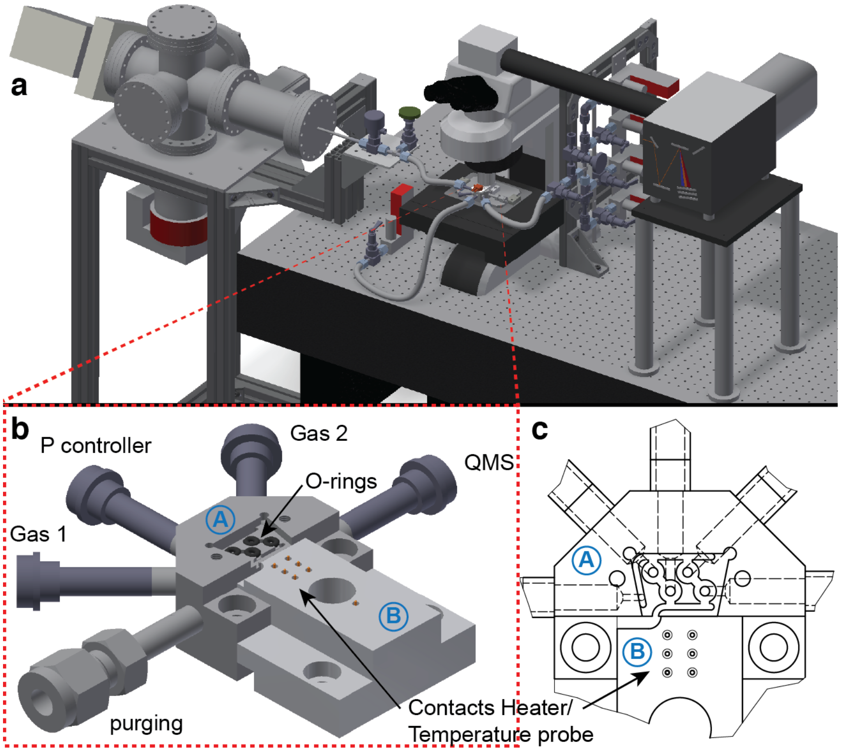

Supplementary Figure 3. Technical drawing of the nanoreactor setup (a) and enlarged picture of the connection block (b,c) that hosts the nanoreactor chip under the microscope. “A” indicates parts manufactured in stainless steel and provides the gas connections to the chip that is pressed against four FPM O-rings. “B” indicates a ceramic block that hosts electrical contacts for resistive heating and a 4 wire RTD sensor are realized with electronic spring pins embedded in the drilled holes.

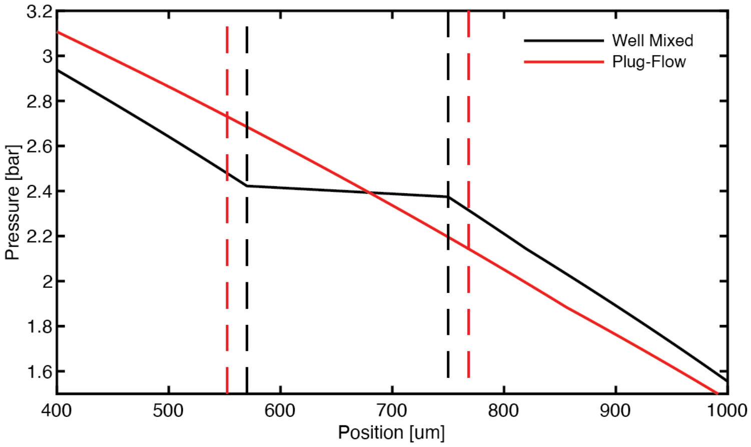

Supplementary Figure 4. Simulated Pressure Profiles. Unified flow model calculations were performed and the resulting pressure profiles for the well-mixed reactor (black) and plug-flow reactor (red) are presented. The dashed lines indicate the regions of each reactor that correspond to the catalyst bed.

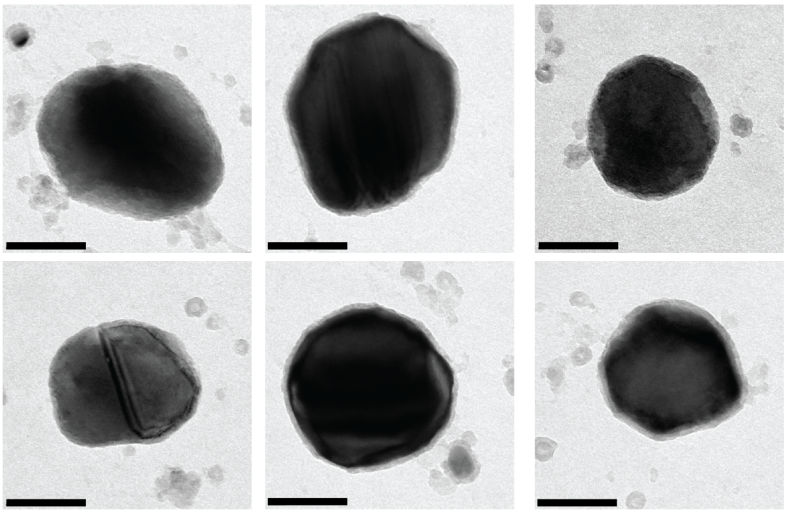

Supplementary Figure 5. TEM of Cu nanoparticles. TEM images of 6 different nominally identical Cu nanoparticles after a cycle of oxidation in 0.5 % O_2, followed by reduction in 7 % CO (both in Ar carrier gas) at 400°C. Scale bar is 50 nm. The shell visible on some of the particle is attributed to the formation of a thin oxide during the transport in air from the reactor to the TEM system.

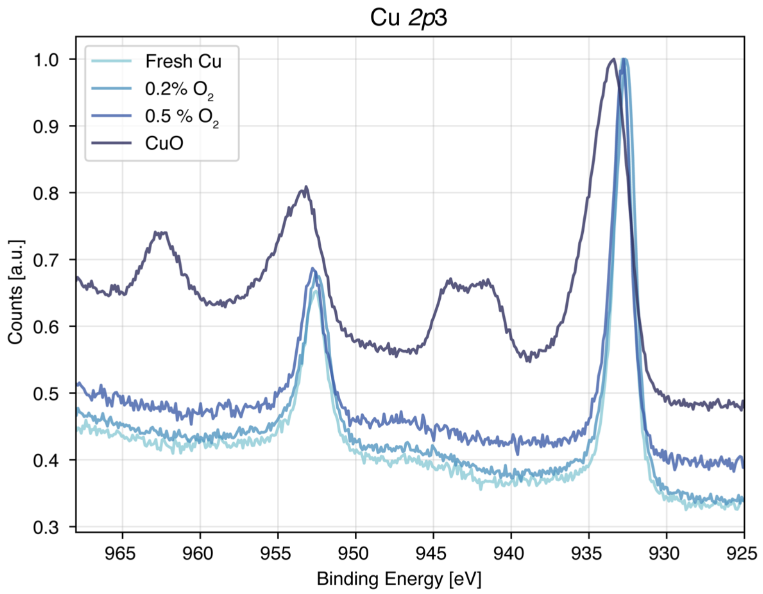

Supplementary Figure 6. XPS of Cu nanoparticles measured after different pretreatment. The dark-blue line (labeled "CuO") corresponds to Cu nanoparticles placed in 1% O_2 in Ar for 1h at 400°C. It exhibits clear satellite peaks at 970 and 942 eV, characteristic for CuO. The three other XPS scans correspond to Cu nanoparticles that have been pre-reduced in 2% H_2 (labeled "fresh Cu") and then exposed to a mix of 7 % CO and 0.2 % O_2 or 0.5 % O_2 , respectively, as indicated in the legend. All samples treated in a CO - O_2 mixture show no indication of CuO formation. Further, the measured Auger parameter of 1849.1eV lets us conclude that they are Cu_2O.[10]

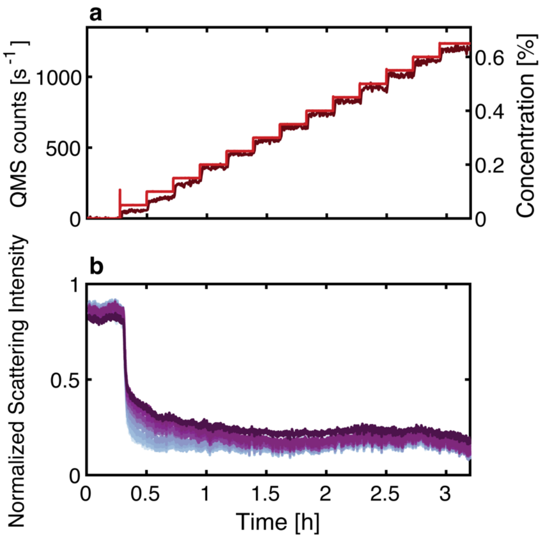

Supplementary Figure 7. Plug-flow reactor exposed to a stepwise increasing O_2 concentration in Ar. a) Set O_2 concentration (red) and measured O_2 counts by the QMS (dark red). b) Optical response from the ten patches showing how they all lose their high scattering intensity simultaneously, as soon as O_2 is introduced.

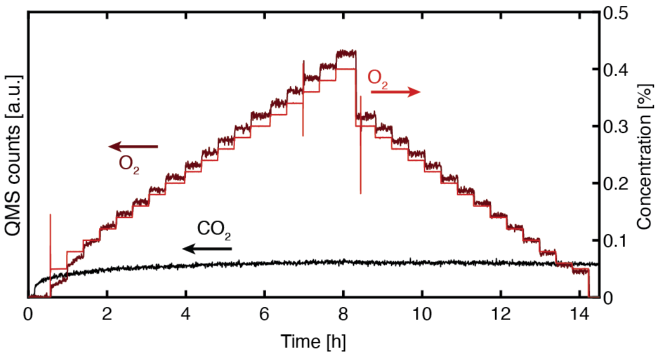

Supplementary Figure 8. Empty reactor control experiment. Measured activity from an empty chip with a design identical to the plug-flow reactor presented in the main text. The experimental procedure was the same as presented in Figure 5 in the main text with a constant CO concentration of 7 % and a stepwise increasing O_2 concentration, all in Ar carrier gas. No measurable CO_2 is produced (black line), confirming the negligible activity of the reactor itself.

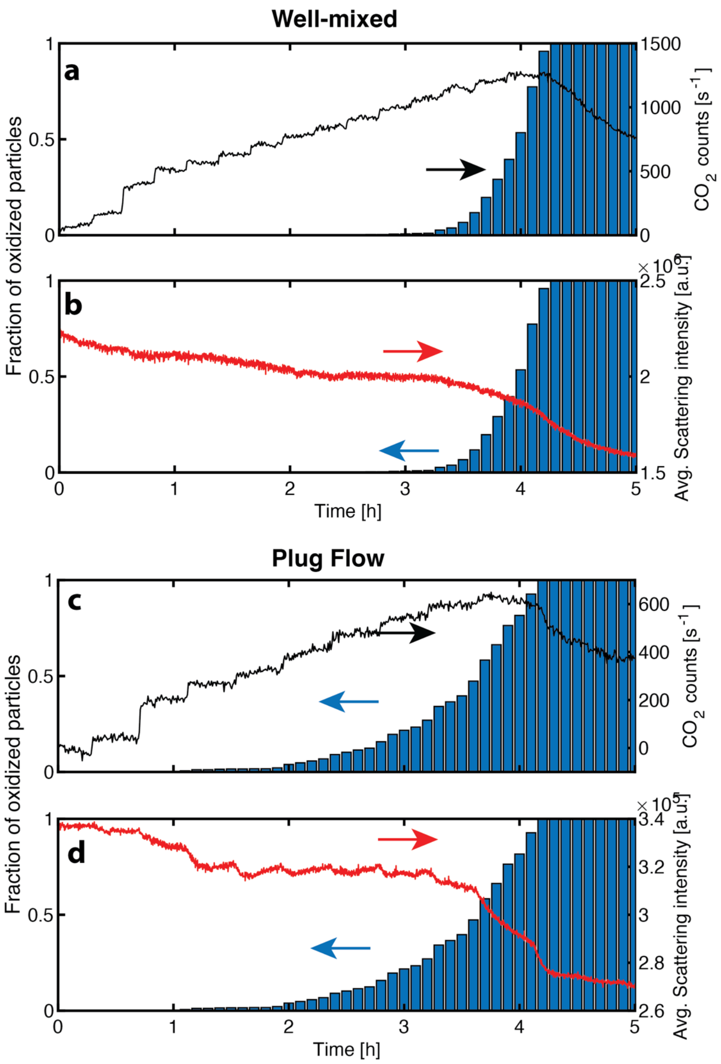

Supplementary Figure 9. Fraction of oxidized particles. The fraction of oxidized particles as a function of time for the well-mixed reactor (a,b) and the plug-flow reactor (c,d). Blue bars show the fraction of oxidized particles (left axis). The black line (a,c) corresponds to the amount of CO_2 measured at the outlet of the reactor and the red lines in (b,d) show the average scattering intensity from all particles.

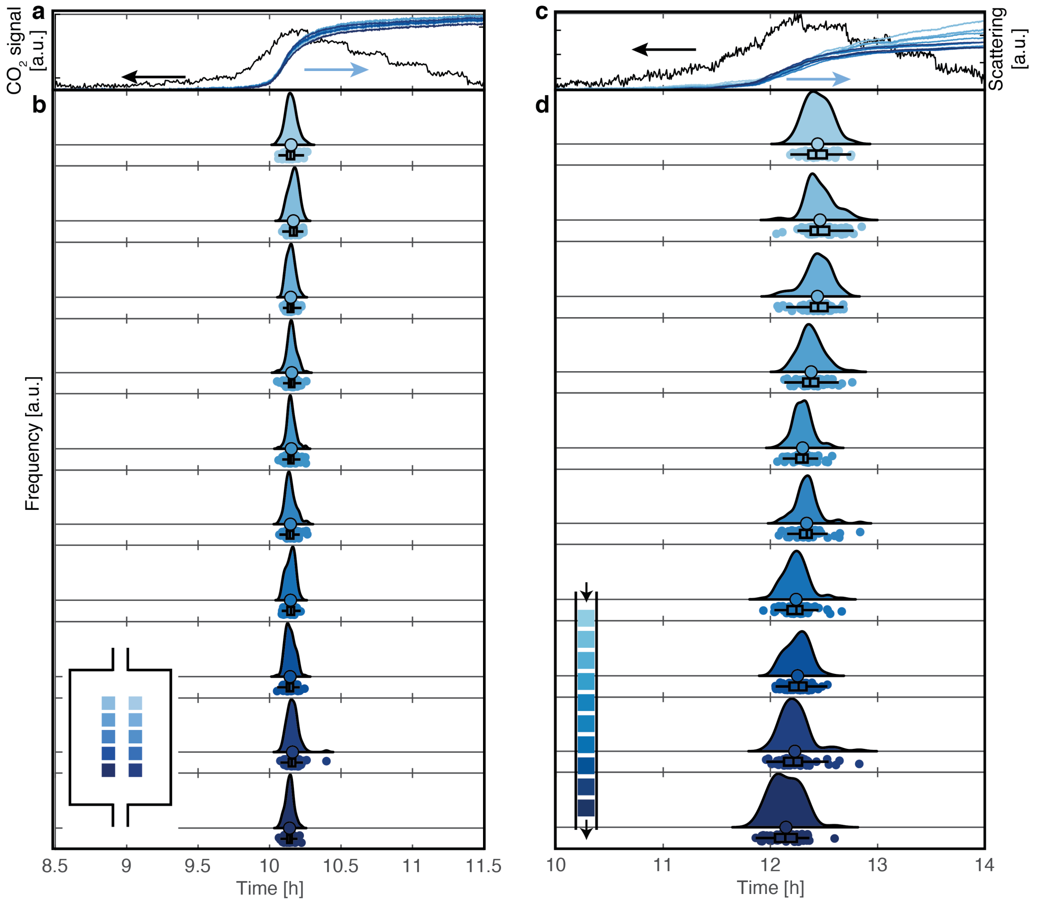

Supplementary Figure 10. Reduction time analysis for the well-mixed (a,b) and the plug-flow (c,d) reactors. a) CO_2 production measured by the QMS at the outlet of the batch reactor (black, left axis) and the scattering intensity from the 10 patches (blue, right axis). b) Reduction times for the individual particles in the well-mixed reactor, presented as a violin plot. Each distribution corresponds to data collected from 100 particles contained within a single array, with each array outlined and color-coded in the inset. Below each of the distributions are the individual particle data points presented as colored dots, the mean oxidation time (outlined large circle) and a box plot showing the median oxidation time, the inner percentile (box) and whiskers corresponding to the lower/upper adjacent values (horizontal line). Inset shows a schematic of the reactor geometry with the particle arrays in different shades of blue. (c,d) Same as (a,b) but for the plug-flow reactor, note the difference in time scale on the x-axis. Here the last patch is reduced first. We can rationalize this by remembering that the O_2 concentration is decreased along the channel and thus the threshold O_2 concentration will be reched for the most down stream patch first.

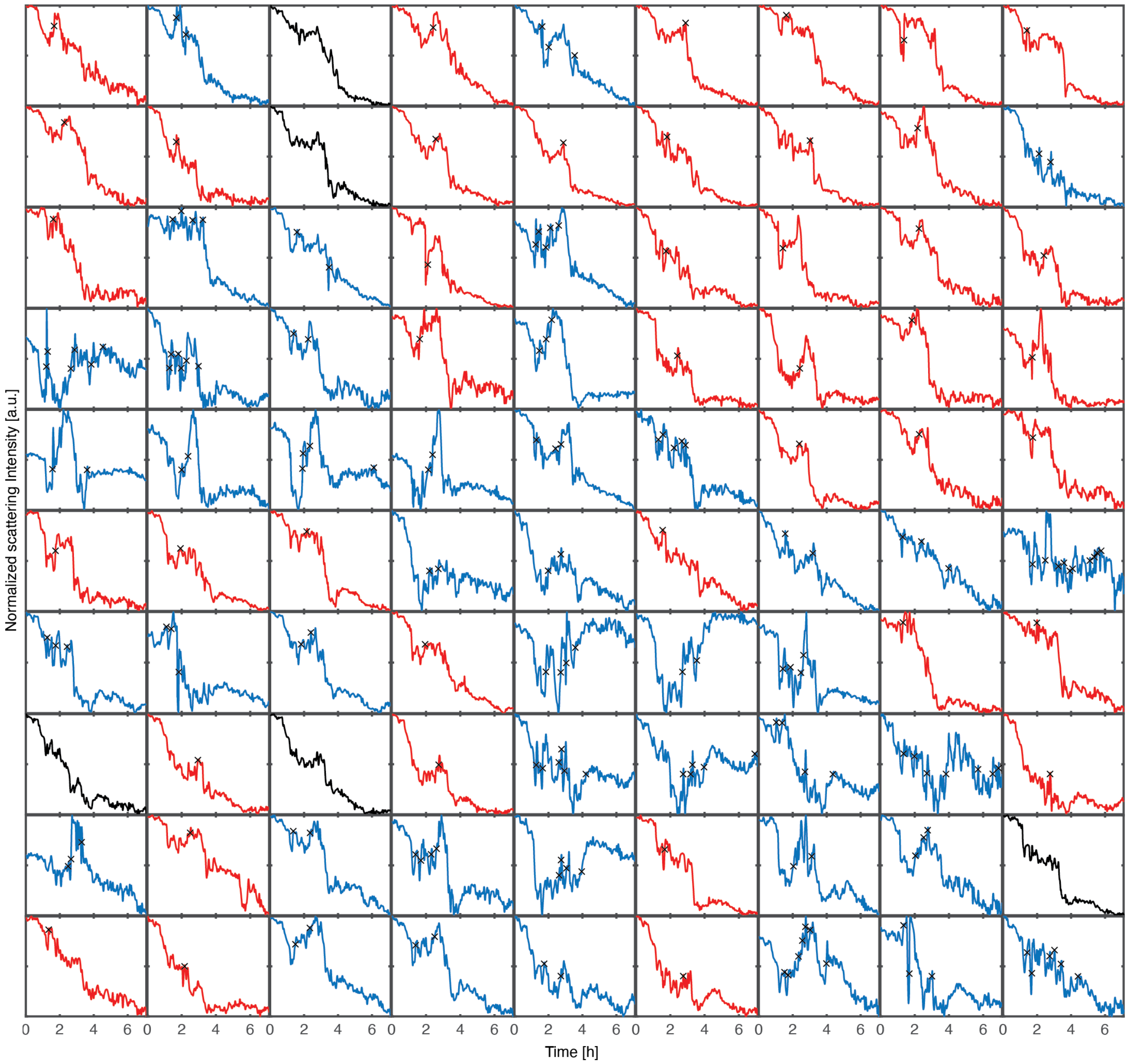

Supplementary Figure 11. Single particle response from representative patch. Single particle scattering intensity from 90 particles collected from "Patch 3" in the plug-flow reactor (cf. **Figure 2b** for patch position in reactor). Traces colored black correspond to particles that do not exhibit a significant recovery of scattering intensity, red traces correspond to traces with one significant intensity recovery and blue traces have > 1 period of significant intensity recovery. Black crosses indicate where each intensity has recovered by 20 %, which we have defined as the threshold to count a particle as recovering.

Supplementary References

1. Albinsson, D. *et al.* Operando detection of single nanoparticle activity dynamics inside a model pore catalyst material. *Sci. Adv.* **6**, eaba7678 (2020).

2. Beskok, A. & Karniadakis, G. E. Report: A model for flows in channels, pipes, and ducts at micro and nano scales. *Microscale Thermophys. Eng.* **3**, 43–77 (1999).

3. Filot, I. A. W. Introduction to microkinetic modeling. (2018).

4. Falsig, H. *et al.* Trends in the Catalytic CO Oxidation Activity of Nanoparticles. *Angew. Chemie Int. Ed.* **47**, 4835–4839 (2008).

5. Zhdanov, V. P. & Kasemo, B. Simulation of kinetic oscillations in the CO+O2/Pt reaction on the nm scale. *J. Catal.* **214**, 121–129 (2003).

6. Olivo-Marin, J.-C. Extraction of spots in biological images using multiscale products. *Pattern Recognit.* **35**, 1989–1996 (2002).

7. Barroo, C., Wang, Z.-J., Schlögl, R. & Willinger, M.-G. Imaging the dynamics of catalysed surface reactions by in situ scanning electron microscopy. *Nat. Catal.* **3**, 30–39 (2020).

8. Jørgensen, M. & Grönbeck, H. The Site-Assembly Determines Catalytic Activity of Nanoparticles. *Angew. Chemie Int. Ed.* **57**, 5086–5089 (2018).

9. Alekseeva, S. *et al.* Grain boundary mediated hydriding phase transformations in individual polycrystalline metal nanoparticles. *Nat. Commun.* **8**, 1084 (2017).

10. Strohmeier, B. R., Levden, D. E., Field, R. S. & Hercules, D. M. Surface spectroscopic characterization of CuAl2O3 catalysts. *J. Catal.* **94**, 514–530 (1985).
