# Supplementary material for: Copper catalysis at operando conditions—bridging the gap between single nanoparticle probing and catalyst-bed-averaging
Source: Nat Commun. 2020 Sep 24;11:4832. doi: 10.1038/s41467-020-18623-1 (PMC7518423; doi:10.1038/s41467-020-18623-1)
Supplement: Supplementary file 3 — Description of Additional Supplementary Files [file 41467_2020_18623_MOESM3_ESM.pdf]

Description of Additional Supplementary Files

File Name: Supplementary Movie 1
Description: Change in scattering intensity for all ten patches in the plug-flow reactor. The first and second row correspond to patch 1-5 and 6-10 in Figure 2b, respectively. Color scale corresponds to scattering intensity difference compared to the first frame of the measurement, ΔScattering(t)=I_xy (t)-I_xy (t=0). Note the blinking particles during the catalytically active time period. For reference cf. Figures 5 & 6d.
